# Anti-diabetic activity of different oils through their effect on arylsulfatases

**DOI:** 10.1186/s40200-014-0116-z

**Published:** 2014-12-09

**Authors:** Rima Samarji, Mahmoud Balbaa

**Affiliations:** Department of Biological & Environmental Sciences, Faculty of Science, Beirut Arab University, Beirut, Lebanon; Department of Biochemistry, Faculty of Science, Alexandria University, Alexandria, Egypt

**Keywords:** Diabetes, Arylsulfatases, Kinetic parameters, Catalase, Oxidative stress

## Abstract

**Background:**

Diabetes mellitus (DM) is characterized by the overproduction of the reactive oxygen species which affects the integrity of the lysosomal membrane affecting lysosomal enzymes. The effect of these species is blocked by some natural products as antioxidants. In the current study, groups of normal and streptozotocin (STZ)-induced diabetic rats were treated by Nigella sativa (NS), olive and canola oils and subjected to the study of arylsulfatases as a model of lysosomal enzymes. The aim of the present study is to investigate the effect of STZ-induced diabetes on arylsulfatases in presence and absence of NS, olive and canola oils.

**Methods:**

Different groups of rats were induced by STZ, treated with different oils and compared to their corresponding control group. All groups were subjected for the assays of blood glucose, insulin, catalase and arylsulfatases. A comparative kinetic study of arylsulfatses was performed to detect the alteration of catalytic characterization.

**Results:**

The results demonstrated that diabetes causes a significant elevation in the level of hepatic arylsulfatase B and a significant reduction of hepatic catalase as an antioxidant enzyme. NS and olive oils returned catalase and arylsulfatase B activities back near to normal by fixing their catalytic properties. Furthermore, the maximum velocity of arylsulfatases A and B was significantly elevated in the induced diabetes, whereas their K_m_ values were significantly changed. The treatment of diabetic rats by NS and olive oils reduced the degree of significance.

**Conclusion:**

Diabetes induces significant alterations of the catalytic characters of arylsulfatases and some oils decrease this alteration through an antioxidant-mediated effect.

## Background

DM is a chronic metabolic disorder that presents a major worldwide health problem. DM is characterized by complete or partial deficiencies in insulin production and/or insulin action coupled with chronic hyperglycemia and disruption in metabolism [1]. In DM, oxidative stress represents an imbalance between the production of free radicals and the ability of the antioxidant defense mechanisms to detoxify them [2,3]. On the other hand, STZ is an antibiotic produced by Streptomycetes achromogenes and is used to stimulate insulin-dependent DM [4].

ASA (EC 3.1.6.8) and ASB (EC 3.1.6.9) are lysosomal enzymes, which are involved in the metabolism of glycoconjugates. ASA is known as cerebroside-3-sulfohydrolase and it desulfates the galactose-3-sulfate residues in cerebroside sulfate and other sulfated galactolipids [5]. An increase in the enzyme concentration was found in the body fluids of patients with cancer of various organs [6]. ASB desulfates the non-reducing terminal N-acetylgalactosamine-4-sulfate residues that are present in glycosaminoglycan [7]. ASB activity is increased in different tumors tumors [5,8-11]. DM is characterized by the overproduction of ROS which affects the lysosomal membrane integrity [12]. ROS were found to decline on increasing the antioxidant enzymes [13] and antioxidant activity of some natural products [14]. It was found that antioxidant therapy can be subdivided into vitamins and supplements, plants and their active ingredients and drugs with antioxidant potential [14-18]. Also, it was found a diverse effect of different oils such as Nigela sativa [19,20] and olive [21-25] oils in DM. It was reported that both NS and olive oils have antidiabetic and antioxidant activities [19,21,26,27]. Taken together, the present research is conducted to investigate the effect of STZ-induced diabetes on arylsulfatases in presence and absence of NS, olive and canola oils. The enzymatic activity of arylsulfatases is assayed in both liver and serum in all groups and the catalytic parameters of ASA and ASB were determined to detect any induced catalytic change of these enzymes in diabetes.

## Materials and methods

### Experimental animals

Sixty four male albino Sprague–Dawley rats of 4–6 weeks old with an approximate body mass of 200 g were obtained from the animal house of the Faculty of Science, Beirut Arab University. Animals were housed in a poly carbonate cages (8 animals/cage) under standardized laboratory conditions with a twelve hour day –night cycle, temperature of 25°C, and humidity of 45% to 60%. Animals had access to tap water and to standard food diet ad libitum. The animal protocols were approved by the regional ethical committee (BAU Ethics Committee).

### Chemicals and oils

STZ, DE-52, insulin ELISA kit and *p*-NCS salt were purchased from Sigma-Aldrich (Beirut office Lebanon). *p*-NCS was prepared as a substrate reagent for ASA ASB as previously explained [28]. CAT assay kit was purchased from Biodiagnostic, Diagnostic and Research Reagents, Giza, Egypt.

### Rats treatment

Eight groups of male rats were prepared, each includes 8 rats. Four of these groups were treated with STZ to induce diabetes in them at a dosage of 50 mg/kg as previously described [4]. Some groups were treated by NS or olive or canola oils. The different groups were classified as non-diabetic rats (control), STZ-induced diabetic rats (GI), control treated with NS oil (GII), diabetic treated with NS oil (GIII), control treated with Olive oil (GIV), diabetic treated with Olive oil (GV), control treated with canola oil (GVI) and diabetic treated with canola oil (GVII). A dose of 0.2 ml/kg/day of NS, olive and canola oils were given daily by i.p injection for 4 weeks.

### Blood Glucose assay

The blood glucose level was measured 3 days and 4 weeks of STZ-PI and oil treatment, respectively. Glucose levels in all groups were measured by means of a glucometer by using accu-check strip and the detected color reflects the glucose concentration in mg/dl [29].

### Insulin assay

Insulin is assayed in the pancreatic homogenate of animals of all groups by Sigma-Aldrich insulin ELISA kit.

### Sampling

Oils treatment occurred within 4 weeks, after which the rats were sacrificed, blood samples collected by cardiac puncture and centrifuged within heparinized tubes at 3000 r.p.m for 20 min to obtain serum samples. Both serum and liver were collected and stored at −80°C until use. The removed hepatic tissues are homogenized in 9 volumes of ice-cold 10 mM Tris–HCl buffer, pH 7.4 containing Triton X-100 and 0.25 M sucrose using a Brinkman homogenizer. The homogenate was centrifuged at 500 × *g* and 4°C for 20 min [30]. The obtained supernatant was subjected to enzymes assay.

### Enzyme assay

CAT activity was assayed as previously described [31]. One unit CAT is defined as that decomposes 1.0 μM of H_2_O_2_/min at 25°C and pH 7. For arylsulfatases, one unit of enzyme activity is defined as one nmol of *p-*nitrocatechol liberated per an hour under these assay conditions [30]. The incubation mixture was incubated at 37°C and the reaction, stopped after 30 min by adding 1 M NaOH and measured for the absorbance at 515 nm.

### Determination of kinetic parameters

The liver homogenate was collected from Control, GI, GIII and GV and then subjected for purification for the kinetic studies. ASA and ASB were purified as previously described [30]. Time courses of the enzymes were performed to determine the values of initial velocities at different substrate concentration ranges (2.0 – 10.0 mM and 10–50 mM for ASA and ASB, respectively) at the above mentioned assay conditions. The values of K_m_ and V_max_ of ASA and ASB were determined from Lineweaver-Burk plots.

### Protein assay

The protein is assayed as described previously [32] using bovine serum albumin as a standard.

### Statistical analyses

All the data are represented as mean ± S.E. The statistical analysis was carried out using the student’s *t*-test (SPSS version 16). All experiments were run on three occasions for reproducibility, and all assays were done in triplicate. Values were considered statistically significant when p < 0.05.

## Results

**Figure 1:**
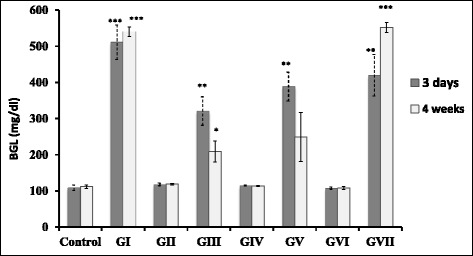
**Blood glucose levels in the different groups 3 days and 4 weeks PI of STZ and oil treatment.** Significant difference for all groups is compared to the control represented by *p < 0.05, **p < 0.01, ***p < 0.001 and non-significant at p > 0.05.

STZ injection induces diabetes leading to an increase in BGL more than 120 mg/dl. BGLs were measured in the different groups 3 days PI of STZ/oil treatment and 4 weeks PI of STZ/oil treatment (Figure 1), respectively. All data were compared to the control group as a reference group. The control group showed a BGL range of 109 ± 7 - 112 ± 5 mg/dl. BGL in GI group showed 4.7 and 4.8 fold - increases, respectively with a significant increase (p < 0.001) compared to the control. BGL in GII, GIV and GVI groups showed a non-significant change (p > 0.05) compared to the control. BGL in GIII, GV and GVII groups showed a significant increase compared to the control (p < 0.05). Insulin level was measured in the pancreatic tissues to assess the effect of the oil treatment on damaged pancreatic tissues in STZ-treated rats. The diabetic group (GI) showed a significant decrease (4.0 fold) in insulin compared to control (p < 0.001). Insulin level showed a non-significant change in GII, GIV and GVI groups (p > 0.05) and a significant increase in GIII, GV and GVII groups (p < 0.05) compared to the control.

**Figure 2:**
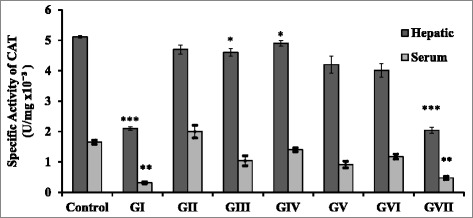
**Specific activity of hepatic and serum CAT (U/mg) 4 weeks PI of STZ and oil treatment.** Significant difference for all groups is compared to the control represented by *p < 0.05, **p < 0.01, ***p < 0.001 and non-significant at p > 0.05.

As shown in Figure 2, the specific activity of CAT in the eight groups is illustrated for the liver tissue and serum, respectively. The enzyme in control group showed specific activities of (5.1 ± 0.04) × 10^−3^ and (1.652 ± 0.07) × 10^−3^ U/mg, respectively. The enzyme in GI showed a value of (2.1 ± 0.06) × 10^−3^ and (0.31 ± 0.03) × 10^−3^ U/mg, respectively with a significant decrease (p < 0.01) compared to control. The enzyme in GII, GV and GVI groups displayed a non-significant change (p > 0.05) compared to control. In addition, the enzyme in GIII showed a value of (4.6 ± 0.121) × 10^−3^ U/mg with a significant decrease (p = 0.023) and (1.037 ± 0.16) × 10^−3^ U/mg and with a non-significant change (p = 0.119), respectively compared to the control. The enzyme In GIV displayed a significant decrease (p = 0.037) and a non-significant change (p = 0.191), respectively compared to control. The enzyme in GVII group displayed a significant decrease (p < 0.05) compared to control.

**Figure 3:**
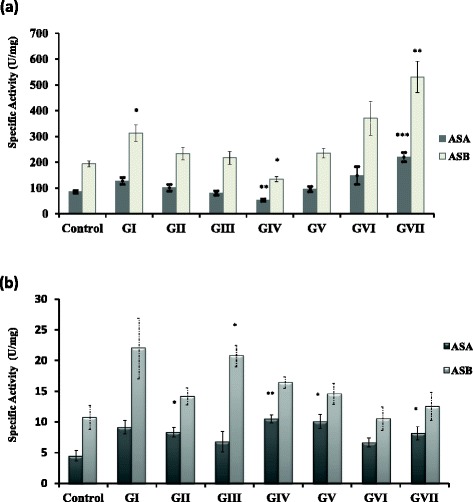
**Specific activities of hepatic (a) and serum (b) ASA & ASB in all groups 4 weeks PI of STZ and oil treatment.** Significant difference for all groups is compared to the control represented by *p < 0.05, **p < 0.01, ***p < 0.001 and non-significant at p > 0.05.

The results in Figure 3a represent the hepatic ASA and ASB specific activity in the eight groups after oil treatment. The control group showed an ASA and ASB specific activity of 85.25 ± 5.14 and 194 ± 11.57 U/mg for ASA and ASB, respectively. ASA and ASB in GI group showed values of 1.5 (non-significant) and 1.6 fold-increase (significant), respectively compared to control, whereas the values in GII group were non-significantly changed compared to the control. The enzymes in GIII, GV and GVI groups showed a non-significant change of compared to the control, whereas the values in GIV group were non-significantly decreased compared to control. The enzymes in GVII showed a significant increase compared to control.

For serum ASA and ASB, the control group showed an ASA and ASB specific activity of 4.5 ± 0.86 and 10.76 ± 1.975 U/mg, respectively. The enzymes in GI and GVI groups displayed a non-significant change compared to control. ASA and ASB in GII group displayed a significant increase and a non-significant change, respectively with respect to control. These enzymes in GIII group displayed a non-significant change and a significant increase, respectively compared to control. In GIV, GV and GVII groups, the enzymes displayed a significant increase and a non-significant change, respectively compared to control (Figure 3b).

**Figure 4:**
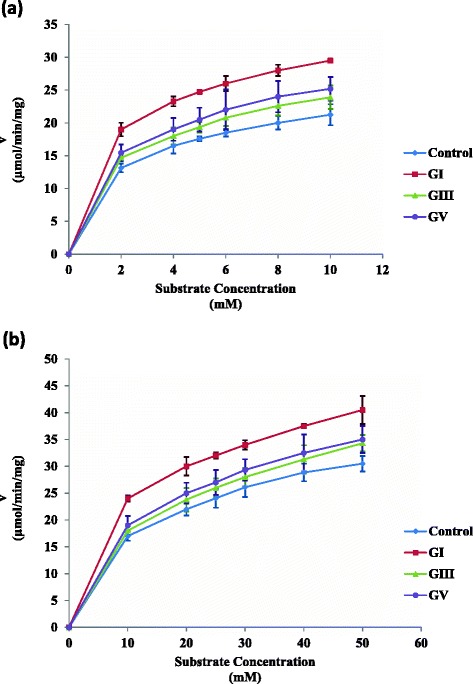
**Effect of different substrate concentrations on the initial velocities of hepatic ASA (a) and ASB (b) from the control, GI, GIII and GV groups.**

The initial velocity versus substrate concentrations for ASA and ASB from the investigated group showed a hyperbolic behavior and the initial rates of these enzymes increase on elevating the substrate concentrations (Figure 4 a & b). The values of the kinetic constants V_max_ and K_m_ of arylsulfatases increased after the induction of diabetes. These values are summarized in Table 1 for the hepatic enzymes from control and treated rats. The purified ASA from the control group shows a V_max_ value of 22.8 ± 1.03 nmol/min/mg protein and a K_m_ value of 1.68 ± 0.011 mM. The enzyme from GI group shows a significant increase of V_max_ value and a significant decrease of K_m_ value compared to control. The enzyme from GIII and GV groups shows a non-significant increase of V_max_ and K_m_ values compared to control. The purified ASB from the control group shows a V_max_ value of 33.07 ± 0.33 nmol/min/mg protein and a K_m_ value of 11.373 ± 0.089 mM. The enzyme from GI, GIII and GV groups shows significance increase (p < 0.05) of V_max_ and K_m_ values compared to control.

**Table 1:** **Kinetic parameters of ASA and ASB from control, GI, GIII and GV where the whole parameters are compared to control and GI groups**

**Enzymes**	**Km**				**Vmax**			
	**Control**	**GI**	**GIII**	**GV**	**Control**	**GI**	**GIII**	**GV**
**ASA**	**1.68 ± 0.01**	**1.48 ± 0.008 b**	**1.72 ± 0.015 y**	**1.768 ± 0.01 y**	**22.8 ± 1.03**	**29.5 ± 0.29 a**	**25.66 ± 0.88**	**27.3 ± 1.06**
**ASB**	**11.37 ± 0.089**	**8.5 ± 0.28 b**	**13.1 ± 0.208 b,z**	**12.37 ± 0.186 a,z**	**33.33 ± 0.33**	**45.15 ± 0.15 c**	**39.66 ± 0.66 a,x**	**39.75 ± 0.75 a,x**

Superscript letters represented by a, b & c indicate a significant variation compared to control group and x, y & z compared to diabetic group at p < 0.05, p < 0.01 and p < 0.001, respectively. No superscript letter is an indication for non-significant variation at p > 0.05. Vmax is expressed in nmol/min/mg and Km is expressed in mM.

## Discussion

A significant elevation in BGL in the diabetic rats compared to control was reported [33]. The BGL in GV group showed a decrease that is still non-significantly increased compared to that of control group. This implies that some oils were useful in improving BGL in DM. Similar studies showed a significant decrease in BGL after olive [21] and NS oils treatment [33] in diabetic rats. The decrease in BGL in NS oil-treated diabetic rats may be attributed to a decrease of gluconeogenesis in liver [34,35]. It is well known that prolonged hyperglycemia favors the permanence of excessive hepatic glucose output due to an increase of liver gluconeogenesis in DM caused by over expression of glucose-6-phosphatase enzyme [36]. The canola oil treatment was deleterious in which a significant increase in BGL of GVII group occurs compared to the control group despite its health benefits listed previously [37,38]. It was found that canola oil-rich diets cause insulin resistance thus favoring type 2 DM and omega-6 fatty acid which is one of the important content of canola was confirmed to mediate type 2 DM [39,40].

The results of the current study showed that hepatic and serum CAT activity in oil-untreated diabetic rats decreases significantly compared to the control group. CAT activity in hepatic tissue of GIII group has decreased significantly compared to the control group, while that of GV showed a non-significant decrease compared to the control group. CAT activity in sera for each of GIII and GV was non-significantly decreased compared to the control group but their activities were greater than that of GI group. This is an evidence that both oils improve the antioxidant defense mechanisms. In an agreement of these results, it was found that NS seed extract has antioxidant activity due to the flavonoids which quenchers O_2_^**-.**^ [41]. Also, NS seed extract-treated diabetic rats showed a significant increase in the hepatic and renal CAT activity [41]. Moreover, it was confirmed that NS oil might play a role against the enzymatic alterations in the liver caused by *Schistosoma mansoni* infection by improving the host immunity due to the NS oil antioxidant activity [27]. However, it was found that the olive oil hydrophilic fraction has an antioxidant potential and a significant increase in the hepatic CAT activity in rats subjected to herbicide treatment [42]. Another study also suggests that the olive oil phenolic compounds have an antioxidant activity [26]. Therefore, we suggest that the effect of NS and olive oils in the current study may be attributed to their antioxidant action. On the other hand, canola oil was not able to correct the oxidative stress produced in DM. A reported study that agrees to this finding reveals that the canola oil decreases the antioxidant status in hypertensive rats [43].

It was previously reported that STZ-induced diabetes has an effect on the cellular glycosaminoglycan (GAG) content and STZ-induced diabetes alters the synthesis and catabolism of GAG [44]. Also, it was found that the sulfation of heparan sulfate is reduced in the liver of STZ-induced diabetic rats [45]. It was reported that sulfatides are required for normal insulin secretion [46] and the promotion of proinsulin folding [47]. Moreover, it was proven that sulfatides reduce the incidence of diabetes in non-obese diabetic mice compared to control [48]. These findings support the data of ASA and ASB in the present study. Accordingly, the elevation of both hepatic ASA and ASB may be attributed to the alteration of the modification of GAG sulfation and sulfatides content, respectively.

In fact, lysosomal membrane permeability occurs in cell death process which also favors the amplification of the death signaling. One of the essential factors that contribute in the lysosomal membrane permeability is ROS formation. In response to the increased ROS, increased amounts of H_2_O_2_ can penetrate into lysosome. The acidic environment of the lysosomal lumen enhances the reduction of iron and the generation of OH^•^ that stimulates the peroxidation of membrane lipids leading to the outflow of lysosomal constituents into cytosol [49]. Therefore, it could be suggested that the elevation in the hepatic ASA and ASB activities may be related to lysosomal membrane permeabilization mediated by oxidative stress, which is one of the predominating features in DM.

The determination of the V_max_ and K_m_ for each of ASA and ASB allows the demonstration of the catalytic alterations in these enzymes that occur under the pathological circumstances. Since the kinetic behavior of both ASA and ASB is hyperbolic in Figure 4, these enzymes have the normal kinetic behavior and do not display the allosteric characterization [50]. The K_m_ values for ASA and ASB from the diabetic group showed a significant decrease in comparison to that of the control group suggesting that there is a change in the substrate affinity for the enzyme [50-52]. It was found previously that a slight change in the ASA affinity results from *Schistosomiasis* and this paralleled the non-significant change of the activity [12]. Accordingly, the obtained data suggests that the significant changes in the activity that occur in diabetes are related to the alterations in the catalytic site and binding affinity for both ASA and ASB. It was previously reported that ASA and ASB are modified by sialylation and phosphorylation in some tumors [53-55] and ASB activity was changed due to its phosphorylated by cAMP-dependent protein kinase in lung cancer [9,10]. Accordingly, we suggest a regulatory mechanism may be involved in diabetes and may lead to a changed behavior of arylsulfatases.

## Conclusion

The present work demonstrates that both NS and olive oils are capable of improving the diabetic complications at the enzymatic level. Their mechanistic action was suggested to be attributed to an antioxidant activity. Conversely, canola oil showed an adverse effect in relation to the diabetic complications and enzymatic alterations.

## Abbreviations

DM: Diabetes mellitus
CAT: Catalase
ASA: Arylsulfatase A
ASB: Arylsulfatase B
NS: *Nigella sativa*
STZ: Streptozotocin
*p*-NCS: *p*-nitrocatechol sulfate
ROS: Reactive oxygen species
PI: Post injection
BGL: Blood glucose level
